# Exhaustion of Activated CD8 T Cells Predicts Disease Progression in Primary HIV-1 Infection

**DOI:** 10.1371/journal.ppat.1005661

**Published:** 2016-07-14

**Authors:** Matthias Hoffmann, Nikos Pantazis, Genevieve E. Martin, Stephen Hickling, Jacob Hurst, Jodi Meyerowitz, Christian B. Willberg, Nicola Robinson, Helen Brown, Martin Fisher, Sabine Kinloch, Abdel Babiker, Jonathan Weber, Nneka Nwokolo, Julie Fox, Sarah Fidler, Rodney Phillips, John Frater, SPARTAC and CHERUB Investigators

**Affiliations:** 1 Peter Medawar Building for Pathogen Research, Nuffield Department of Medicine, Oxford, United Kingdom; 2 Division of Infectious Diseases and Hospital Epidemiology, Kantonsspital, St. Gallen, Switzerland; 3 Department of Hygiene, Epidemiology & Medical Statistics, Athens University Medical School, Athens, Greece; 4 MRC Clinical Trials Unit at UCL Institute of Clinical Trials & Methodology, London, United Kingdom; 5 The Oxford Martin School, Oxford, United Kingdom; 6 Oxford National Institute of Health Research Biomedical Research Centre, Oxford, United Kingdom; 7 Brighton and Sussex University Hospitals, Brighton, United Kingdom; 8 Division of Infection and Immunity, University College London, London, United Kingdom; 9 Division of Medicine, Wright Fleming Institute, Imperial College, London, United Kingdom; 10 Chelsea and Westminster Hospital, London, United Kingdom; 11 Department of Genitourinary Medicine and Infectious Disease, Guys and St Thomas' NHS Trust, London, United Kingdom; University of North Carolina at Chapel Hill, UNITED STATES

## Abstract

The rate at which HIV-1 infected individuals progress to AIDS is highly variable and impacted by T cell immunity. CD8 T cell inhibitory molecules are up-regulated in HIV-1 infection and associate with immune dysfunction. We evaluated participants (n = 122) recruited to the SPARTAC randomised clinical trial to determine whether CD8 T cell exhaustion markers PD-1, Lag-3 and Tim-3 were associated with immune activation and disease progression. Expression of PD-1, Tim-3, Lag-3 and CD38 on CD8 T cells from the closest pre-therapy time-point to seroconversion was measured by flow cytometry, and correlated with surrogate markers of HIV-1 disease (HIV-1 plasma viral load (pVL) and CD4 T cell count) and the trial endpoint (time to CD4 count <350 cells/μl or initiation of antiretroviral therapy). To explore the functional significance of these markers, co-expression of Eomes, T-bet and CD39 was assessed. Expression of PD-1 on CD8 and CD38 CD8 T cells correlated with pVL and CD4 count at baseline, and predicted time to the trial endpoint. Lag-3 expression was associated with pVL but not CD4 count. For all exhaustion markers, expression of CD38 on CD8 T cells increased the strength of associations. In Cox models, progression to the trial endpoint was most marked for PD-1/CD38 co-expressing cells, with evidence for a stronger effect within 12 weeks from confirmed diagnosis of PHI. The effect of PD-1 and Lag-3 expression on CD8 T cells retained statistical significance in Cox proportional hazards models including antiretroviral therapy and CD4 count, but not pVL as co-variants. Expression of ‘exhaustion’ or ‘immune checkpoint’ markers in early HIV-1 infection is associated with clinical progression and is impacted by immune activation and the duration of infection. New markers to identify exhausted T cells and novel interventions to reverse exhaustion may inform the development of novel immunotherapeutic approaches.

## Author Summary

After being infected with HIV, the pace of disease progression is highly variable between individuals. Some stay well with functioning immune systems for many years, whilst others progress to AIDS quickly. Understanding the factors that underpin these differences is important and may relate to factors such as viral adaptation and immune exhaustion. Recently, there has been interest in certain molecules—called ‘exhaustion’ or ‘immune checkpoint’ markers—which reflect how well the immune system functions. Recent trials show that therapies directed against these molecules can improve anti-cancer immunity. It is known from laboratory experiments that these markers are abundant in HIV infection suggesting that the human immune response to HIV is not fully effective. The relevance of these markers in patient cohorts remains unclear. This study measures three exhaustion markers—PD-1, Tim-3, Lag-3 –in individuals with HIV recruited to a randomised controlled trial of therapy in early HIV infection called SPARTAC. We find a complex picture in which these markers alone, together and in combination with other markers that reflect T cell activation (CD38) help predict the speed of clinical progression and immune decline, with differing effects dependent on the duration of infection. We propose that therapies directed against these markers could impact disease progression, vaccine efficacy or even newer curative strategies.

## Introduction

Following infection with Human Immunodeficiency Virus Type 1 (HIV-1) the rate at which an individual develops AIDS is highly variable ranging from ‘progressors’ who, if untreated, experience rapid CD4 T cell decline in months to years to ‘elite controllers’, who spontaneously maintain undetectable plasma viraemia, often for decades. The tempo of HIV-1-associated disease progression might rest with particular characteristics of HLA class I molecules and the CD8 T cell immune responses which they dictate [1–5]. When a CD8 T cell encounters its cognate antigen, the up-regulation of T cell inhibitory molecules tightly controls the subsequent T cell activation [6–8], and inhibits autoimmunity [9–11]. However, the persistence of antigen can overcome homeostatic controls and lead to permanent CD8 T cell dysfunction or exhaustion [12–16]. In HIV-1 infection T cell exhaustion is associated with the up-regulation of surface molecules called immune checkpoint receptors (ICRs) such as PD-1, Tim-3 and Lag-3 [12,17–20], which have also been associated with the size of the HIV reservoir and time to viral rebound after therapy cessation[21,22].

We sought to determine whether, in primary HIV-1 infection (PHI), these indicators of CD8 T cell exhaustion would correlate with surrogate markers of disease (e.g. HIV-1 plasma viral load (pVL), CD4 T cell count) and actual time to progression within a strictly defined patient population enrolled into a randomized clinical trial of early antiretroviral therapy (ART). In particular, we wanted to study exhaustion in activated CD38 CD8 positive T cell populations, as CD38 expression has also been correlated with disease progression. We found significant associations between ICR expression and both pVL and disease progression, and an enhanced effect when co-expressed on activated T cells.

## Results

### Analysis of baseline characteristics of SPARTAC participants

366 participants were enrolled into the SPARTAC trial[23]. Of 156 participants recruited at UK sites, 122 had adequate numbers of peripheral blood mononuclear cells (PBMCs) available for analysis at the pre-therapy ‘baseline’–the closest documented visit to the estimated date of seroconversion. Patient characteristics are detailed in Table 1. The median (interquartile range, IQR) age at enrolment was 34 (28, 41) years. Only five participants were female, and the predominant mode for HIV-1 acquisition was sex between men (MSM) (93%). Of the 122, 41 were randomised to receive no immediate ART (SOC), 44 12 weeks ART (ART12) and 37 48 weeks ART (ART48). The median (IQR) time since the estimated date of seroconversion at randomisation was 77 (54, 98) days. The median (IQR) times for participants in ‘early’ (≤12 weeks after seroconversion) and ‘late’ (>12 weeks after seroconversion) were 60 (42, 72) and 104 (93, 122) days, respectively. MSM were significantly over-represented in the early PHI group. The median (IQR) baseline CD4 T cell count and HIV-1 pVL were 550 (435, 675) cells/μl and 4.70 (4.0, 5.3) log_10_ RNA copies/ml, respectively, without significant differences in participants enrolled ≤ or > 12 weeks into PHI.

**Table 1:** Demographic and clinical characteristics of UK participants in the SPARTAC trial included in the analyses.

	Time from seroconversion to baseline measurement		Overall	
	≤12 weeks (n = 72; 59%)	>12 weeks (n = 50; 41%)	(N = 122; 100%)	P value
**Sex**				0.16
* Male*	71 (99)	46 (92)	117 (96)	
* Female*	1 (1)	4 (8)	5 (4)	
**Risk Group**				0.008
* MSM*	70 (97)	43 (86)	113 (93)	
* Heterosexual*	1 (1)	7 (14)	8 (7)	
* Unknown*	1 (1)	0 (0)	1 (1)	
**Age** *(years)*	34.5 (28.5, 40.0)	34.0 (27.0, 42.0)	34.0 (28.0, 41.0)	0.73
**Baseline CD4 T cell count** *(cells/μL)*	540 (433, 675)	560 (446, 675)	550 (435, 675)	0.85
**Baseline HIV-1 RNA** *(log*_*10*_ *copies/mL)*	5.0 (3.9, 5.5)	4.6 (4.2, 5.1)	4.7 (4.0, 5.3)	0.080
**Days from seroconversion to randomization**	60 (42, 73)	104 (93, 122)	77 (54, 98)	

Data are presented for the 122 participants overall, as well as for times from seroconversion estimated to be ≤ or > 12 weeks. Values are N (%) for categorical and median (interquartile range) for continuous variables. P-values determined by Fisher’s exact and Mann-Whitney tests, respectively.

### CD8 T cell exhaustion correlates with HIV-1 plasma viral load and CD4 T cell count

**Fig 1:**
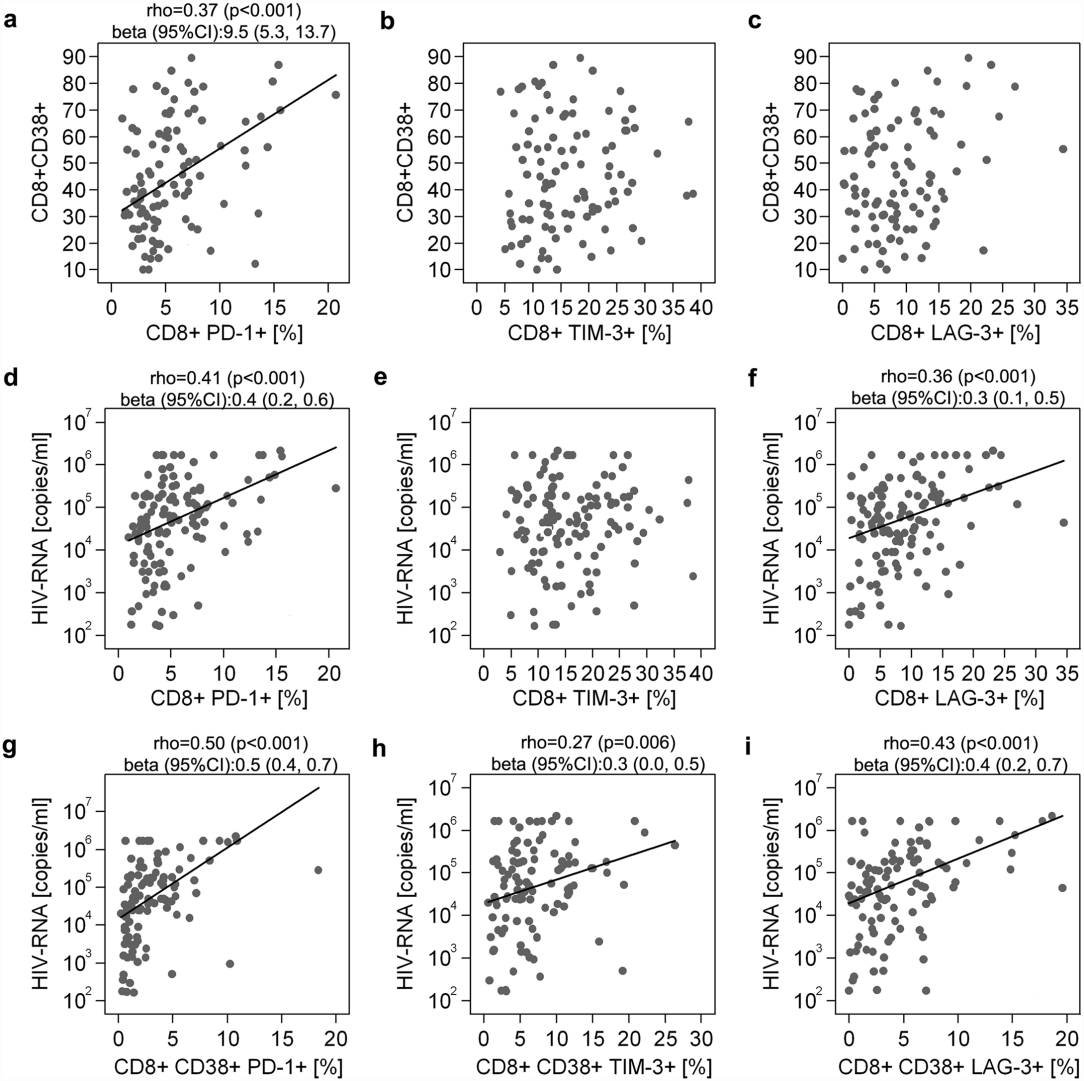
Association between PD-1, Tim-3 or Lag-3 expression on CD8 T cells with CD38 expression and HIV-1 plasma viral load. Expression of PD-1, Tim-3 and Lag-3 expression on CD8 T cells was compared with expression of CD38 on CD8 T cells (a-c). Expression of PD-1, Tim-3 and Lag-3 expression on both CD8 T cells (d-f) and CD8 CD38 T cells (g-i) was correlated with plasma viral load measured at the same time. Associations were evaluated using Spearman correlations. P values and Spearman’s rho are presented. The fitted line, superimposed in the relevant scatterplots, was estimated through linear regression. For significant results, the value of beta (the estimated change in the vertical axis variables (log_10_ scale for HIV-1 RNA) per 1 SD increase in horizontal axis variables) and its confidence intervals (CI) are reported.

The expression of PD-1, Tim-3 and Lag-3 on the surface of CD8 T cells and the subset of activated CD8 T cells that co-expressed CD38 was determined by flow cytometry (Fig A in S1 Text) and was up-regulated in PHI compared with healthy controls (P<0.01 for all comparisons; Mann-Whitney)(Fig B in S1 Text). Expression of CD38 on CD8 T cells correlated with PD-1 expression (Spearman’s rho 0.37; p<0.001) (Fig 1a) but not with Tim-3 or Lag-3 (Fig 1b and 1c). Table 2 shows the percentage of CD8 T cells expressing each of the three exhaustion markers at the pre-therapy baseline time-point; 4.5%, 14.1% and 8.0% of all CD8 T cells and 2.1%, 6.1% and 3.9% of CD38 CD8 T cells (denominator all CD8 T cells) expressed PD-1, Lag-3 and Tim-3, respectively (Table 2). When stratified by time since seroconversion (≤12 weeks or >12 weeks), participants sampled earlier had (with the exception of PD-1 and PD-1/Tim-3 co-expression for which there was a trend) significantly higher percentages of expression of single and dual-expressed markers on CD8 CD38 T cells. The same was not observed for bulk CD8s (Table 2).

**Table 2:** Expression of exhaustion markers on CD8 and CD38 CD8 T cells by time from seroconversion to pre-therapy baseline measurement.

		Time between seroconversion and baseline		
	Overall	< = 12 weeks	>12 weeks	
	Median (IQR)	Median (IQR)	Median (IQR)	P value^+^
**% CD8**				
PD-1	4.5 (2.9, 7.2)	5.0 (3.2, 7.7)	4.2 (2.8, 6.9)	0.17
Tim-3	14.1 (11.1, 20.7)	15.3 (11.6, 20.6)	12.8 (9.3, 21.6)	0.38
Lag-3	8.0 (4.6, 12.5)	8.3 (4.8, 13.5)	7.7 (3.8, 11.3)	0.31
PD-1/Tim-3	0.7 (0.4, 1.3)	0.7 (0.4, 1.5)	0.6 (0.4, 1.1)	0.32
PD-1/Lag-3	0.7 (0.4, 1.1)	0.7 (0.4, 1.1)	0.6 (0.4, 1.0)	0.66
Lag-3/Tim-3	1.6 (0.9, 2.4)	1.6 (0.9, 2.4)	1.7 (0.9, 2.4)	0.99
**% CD8 CD38**				
PD-1	2.1 (1.1, 4.1)	2.7 (1.2, 4.5)	1.8 (0.9, 2.9)	0.054
Tim-3	6.1 (3.4, 10.0)	6.6 (4.6, 10.3)	4.7 (2.5, 9.3)	0.017
Lag-3	3.9 (1.7, 6.8)	4.8 (2.3, 7.2)	2.9 (1.1, 5.9)	0.006
PD-1/Tim-3	0.4 (0.2, 0.9)	0.5 (0.3, 1.1)	0.3 (0.2, 0.5)	0.057
PD-1/Lag-3	0.5 (0.2, 0.8)	0.6 (0.2, 0.9)	0.3 (0.1, 0.6)	0.012
Lag-3/Tim-3	1.0 (0.4, 1.6)	1.1 (0.7, 1.7)	0.8 (0.3, 1.4)	0.021

Percentage surface expression of PD-1, Tim-3 and Lag-3 either on their own or co-expressed. Cells analysed were CD8 T cells and activated CD38+ve CD8 T cells. For all data the denominator is total CD8 T Cells. Data are given as median values plus interquartile range (IQR). P-values were determined by Mann-Whitney tests.

^+^ when corrected for multiple testing overall critical p = 0.004

Correlations between the expression of ICRs on CD8 and CD38 CD8 T cells with CD4 T cell count, pVL and time since seroconversion were assessed (Table 3 and Fig 1d–1i). PD-1 and Lag-3 (Fig 1d and 1f), but not Tim-3 (Fig 1e), expression on CD8 T cells was associated with higher pVL (p<0.001 for both). Expression of ICRs on CD38 CD8 T cells was significantly and more strongly associated with higher pVL compared with on the bulk CD8 positive population (p<0.01 for all markers; Table 3, Fig 1g–1i). Co-expression on CD8 and CD38 CD8 T cells of either PD-1/Tim-3, PD-1/Lag-3 and Tim-3/Lag-3 was also positively associated with pVL (Table 3). PD-1 and PD-1/Lag-3 co-expression on CD8 T cells, and PD-1, PD-1/Tim-3 and PD-1/Lag-3 expression on CD38 CD8 T cells were significantly associated with lower baseline CD4 T cell counts (Table 3). These associations persisted after adjusting for multiple comparisons apart from that between CD4 T cell count and co-expression of PD-1/Lag-3 on CD38 CD4 T cells.

**Table 3:** Correlation of PD-1, Tim-3 and Lag-3 expression on CD8 and CD38 CD8 T cells at baseline with CD4 T cell count, plasma HIV-1 RNA and time since seroconversion.

Marker	Baseline CD4 count	Baseline HIV-1 RNA	Time from seroconversion to baseline
**CD8**			
PD-1	-0.24 (0.008^+^)	0.41 (<0.001*)	-0.14 (0.13)
Tim-3	-0.02 (0.80)	0.05 (0.56)	-0.01 (0.89)
Lag-3	-0.03 (0.75)	0.36 (<0.001*)	-0.16 (0.074)
PD-1/Tim-3	-0.17 (0.072)	0.20 (0.033*)	-0.04 (0.67)
PD-1/Lag-3	-0.24 (0.010^+^)	0.37 (<0.001*)	-0.11 (0.26)
Tim-3/Lag-3	-0.08 (0.37)	0.28 (0.002*)	-0.08 (0.37)
**CD8 CD38**			
PD-1	-0.29 (0.002^+^)	0.50 (<0.001*)	-0.18 (0.061)
Tim-3	-0.16 (0.11)	0.27 (0.006*)	-0.17 (0.093)
Lag-3	-0.10 (0.30)	0.43 (<0.001*)	-0.31 (0.001^†^)
PD-1/Tim-3	-0.28 (0.004^+^)	0.43 (<0.001*)	-0.15 (0.13)
PD-1/Lag-3	-0.21 (0.036)	0.52 (<0.001*)	-0.28 (0.004^†^)
Tim-3/Lag-3	-0.15 (0.12)	0.43 (<0.001*)	-0.23 (0.017)

Percentage surface expression of PD-1, Tim-3 and Lag-3 either on their own or co-expressed. Cells analysed were CD8 T cells and activated CD38 positive CD8 T cells. Results of the correlations with CD4 T cell count, plasma HIV-1 RNA viral load and time since estimated date of seroconversion are given as Spearman's rho (P value). All values give to two decimal places, except for P values <0.1 (3 decimal places).

^+^: significant at the 0.05 level after correction for testing multiple correlations with baseline CD4 T count (corrected overall critical p = 0.017)

*: significant at the 0.05 level after correction for testing multiple correlations with baseline HIV-1-RNA (corrected overall critical p = 0.046)

^†^: significant at the 0.05 level after correction for testing multiple correlations with time from seroconversion to baseline (corrected overall critical p = 0.008)

### Association of exhaustion markers with clinical progression

**Fig 2:**
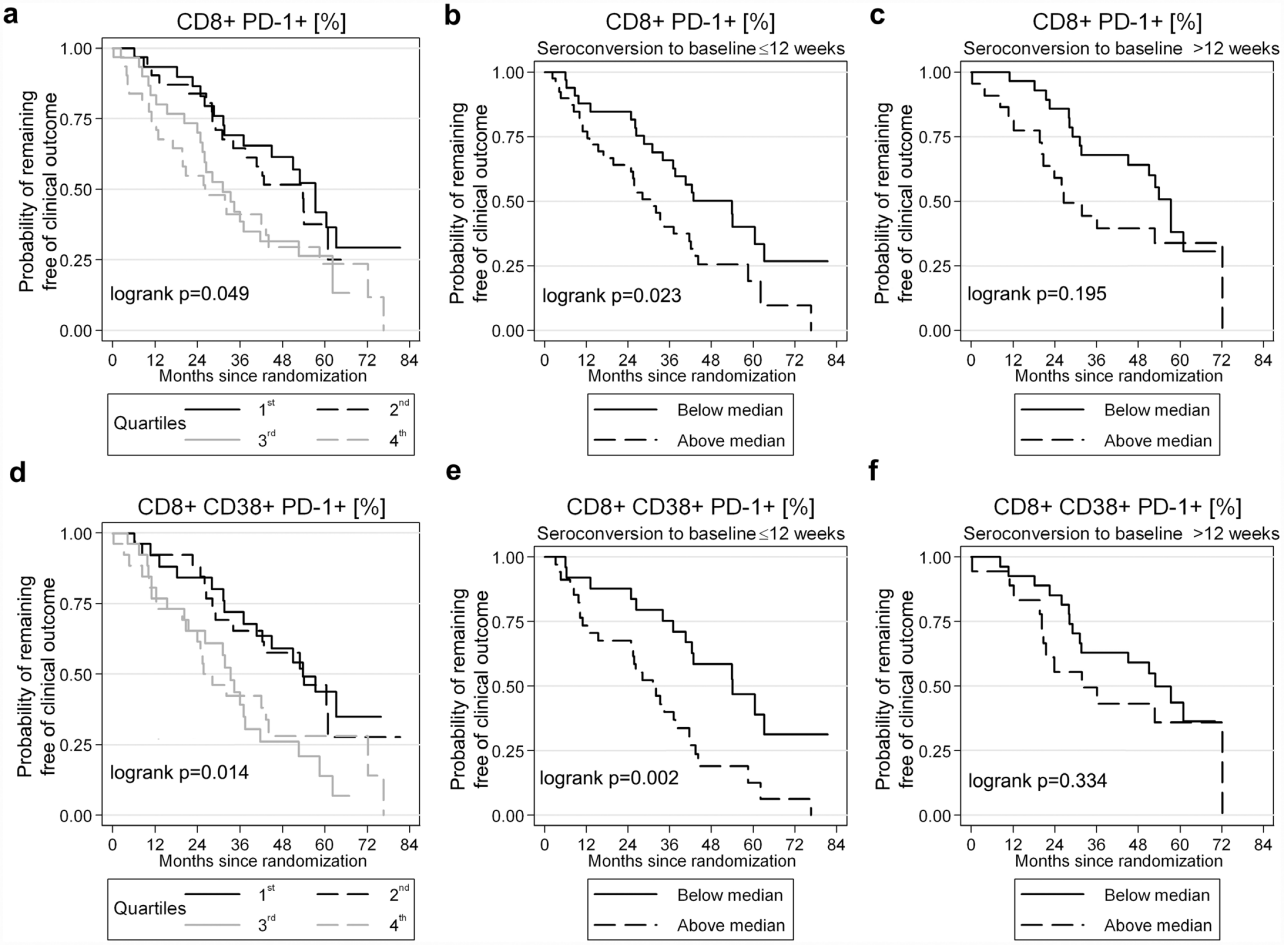
Impact of PD-1 expression on CD8 or CD38 CD8 T cells on clinical outcome. Survival analyses to show the effect of PD-1 expression on (a-c) CD8 and (d-f) CD38 CD8 cells on time to the primary end-point in the SPARTAC trial (CD4 T cell count <350 cells/μl or initiation of long-term ART). In panels a and d, percentage of PD-1 expression is stratifed into quartiles and significance is tested using a log rank test. Survival analyses show the impact of estimated time since seroconversion (reported as ≤12 weeks or >12 weeks) and PD-1 baseline expression on CD8 (b-c) and CD38 CD8 (e-f) T cells. Here, percentage PD-1 expression is stratifed at the median value.

**Fig 3:**
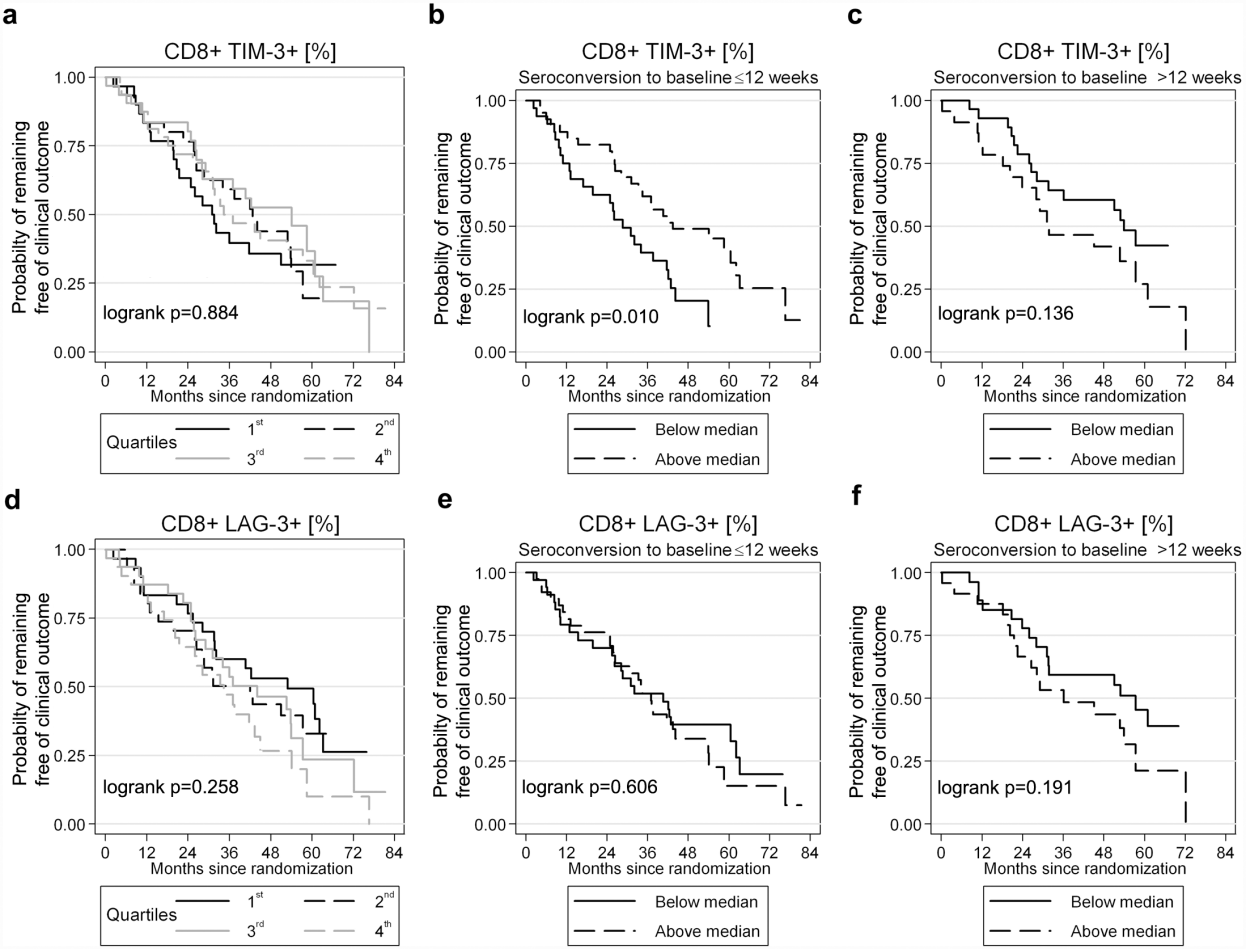
Impact of CD8 T cell Tim-3 and Lag-3 expression on clinical outcome. Survival analyses showing the effect of Tim-3 (a-c) and Lag-3 (d-f) expression on time to the primary end-point in the SPARTAC trial (CD4 T cell count <350 cells/μl or initiation of long-term ART). Percentage of marker expression is stratifed into quartiles (a, d) or, when tested in conjunction with the estimated time since seroconversion (reported as ≤12 weeks (b,e) or >12 weeks (c,f)), at the median value. Significance is tested using a log rank test.

Next we evaluated PD-1, Lag-3 and Tim-3 expression and progression to the SPARTAC trial primary endpoint (either CD4 T cell count <350 cells/μl or (re)initiation of ART). PD-1 expression at baseline on CD8 and CD38 CD8 T cells predicted clinical progression (log rank test, p = 0.049 and p = 0.014, respectively) (Fig 2a and 2d). This effect was most evident in patients recruited within 12 weeks of infection, when compared with those recruited after 12 weeks (Fig 2b and 2e vs 2c and 2f). Tim-3 and Lag-3 expression on CD8 (Fig 3) or CD38 CD8 (Fig C in S1 Text) T cells was not associated with clinical progression when the whole cohort was analysed although, surprisingly, there was evidence for slower disease progression in participants recruited ≤12 weeks from seroconversion with increased Tim-3 expression on CD8 T cells (log rank test, p = 0.01; Fig 3b). This could not be explained through a correlation with Tim-3 expression and PD-1 (or Lag-3) (Table C in S1 Text) and this advantage survived (P = 0.075) adjustment for PD-1, Lag-3, baseline CD4 count and therapy (Table D in S1 Text).

Cox proportional hazards models were used to explore further the association between baseline PD-1 expression on bulk CD8 T cells and clinical progression (Table 4). The association with PD-1 expression and clinical progression was preserved when ART alone (HR 1.75; p = 0.045) or ART and baseline CD4 T cell count (HR 1.76; p = 0.047) were included in the model, but not when accounting for pVL or following correction for multiple comparisons. In a model including time from seroconversion, ART and baseline CD4 T cell count there was a trend for an association between PD-1 expression and clinical progression (HR 1.72, 95% CI 0.98–3.03), but this was not significant when baseline pVL was included. When the magnitude of PD-1 expression was stratified into quartiles, there was evidence of a dose effect (HR: 1, 1.60, 1.71, 2.16 for each quartile, respectively) when ART and baseline CD4 T count were included in the model, but not when baseline pVL was included. We found no evidence to support a significant association with CD38 alone and time to the trial endpoint.

**Table 4:** Cox proportional hazards models on disease progression and PD-1, Tim-3 and Lag-3 expression.

	Unadjusted HR (95% CI; P value^+^)	Adjusted HR (95% CI; P value)		
		ART	ART CD4 T cell count	ART CD4 cell count HIV-1 RNA^‡^
**CD8**				
PD-1	1.68 (0.98, 2.86; 0.057)	1.75 (1.01, 3.01; 0.045)	1.76 (1.01, 3.09; 0.047)	0.99 (0.53, 1.83; 0.96)
Tim-3	0.99 (0.73, 1.33; 0.928)	0.98 (0.73, 1.31; 0.88)	1.00 (0.73, 1.35; 0.98)	0.91 (0.66, 1.25; 0.55)
Lag-3	1.30 (0.95, 1.77; 0.10)	1.35 (1.00, 1.84; 0.054)	1.46 (1.05, 2.04; 0.024^†^)	1.07 (0.73, 1.58; 0.72)
**CD38 CD8**				
PD-1	2.43 (1.16, 5.12; 0.019)	2.71 (1.28, 5.74; 0.009*)	2.06 (0.96, 4.42; 0.064)	0.93 (0.37, 2.33; 0.88)
Tim-3	1.81 (1.14, 2.88; 0.012)	1.87 (1.17, 2.99; 0.009*)	2.05 (1.25, 3.34; 0.004^†^)	1.55 (0.91, 2.65; 0.11)
Lag-3	1.62 (0.99, 2.66; 0.057)	1.84 (1.10, 3.09; 0.020*)	2.06 (1.17, 3.61; 0.012^†^)	1.00 (0.49, 2.03; 1.00)

Cox proportional hazards model showing association with PD-1, Tim-3 and Lag-3 expression on CD8 and CD8 CD38 T cells with the SPARTAC trial primary endpoint. Data are presented unadjusted, and then in multivariable analyses including ART, CD4 T cell count and plasma viral load (HIV-1 RNA). Data are given as (HR (95% CI; p-value).

^+^: corrected for multiple testing overall critical p = 0.008

*: significant at the 0.05 level after correction for testing multiple markers (corrected overall critical p = 0.025)

^†^: significant at the 0.05 level after correction for testing multiple markers (corrected overall critical p = 0.025)

^‡^: corrected for multiple testing overall critical p = 0.008

Cox models also demonstrated an effect of bulk CD8 T cell Lag-3 expression on progression when adjusted for ART initiation and CD4 T cell count (HR 1.46; p = 0.024), but not when including pVL (Table 4). As for PD-1 expression, there were significant associations with clinical progression when including time since seroconversion, ART and baseline CD4 T cell count in the model (HR 1.45; p = 0.03) or when exploring dose effect when stratified into quartiles (HR 1, 1.43, 1.12, 2.17), but neither survived correction for baseline pVL. Tim-3 expression on bulk CD8 T cells did not predict progression.

Interestingly, on restricting the Cox models to CD38 CD8 T cells, all three markers were associated with disease progression in unadjusted models or when correcting for ART and CD4 T cell count although, again, not when adjusting for baseline pVL (Table 4).

### Co-expression of T cell exhaustion markers and clinical progression

Having explored the impact of single exhaustion markers on clinical outcome, we next measured whether their co-expression would predict progression (Fig D in S1 Text and Table A in S1 Text). In survival analyses PD-1/Lag-3 co-expression on CD8 and CD38 CD8 T cells (Fig Da and Dd in S1 Text; log-rank test, p = 0.012 and p = 0.027 respectively), and PD-1/Tim-3 co-expression on CD38 CD8 T cells (Fig Dj in S1 Text, p = 0.01) were associated with faster time to the trial primary endpoint, although not a better predictor than PD-1 alone. When considering time since seroconversion, the effects of PD-1/Lag-3 and PD-1/Tim-3 co-expression on bulk CD8s on clinical progression seemed more pronounced in patients recruited more than 12 weeks after seroconversion, compared with those recruited within 12 weeks (p = 0.030 and p = 0.024, respectively (Fig Dc and Di in S1 Text)). However, when restricting the analyses to activated CD38 CD8 T cells, PD-1/Lag-3 and PD-1/Tim-3 co-expression were, in contrast, associated with progression in individuals recruited within 12 weeks of seroconversion (p = 0.008, p = 0.008, respectively (Fig De and Dk in S1 Text)). There was no effect associated with Tim-3/Lag-3 co-expression.

In Cox proportional hazards models, there was a strong association with high PD-1/Tim-3 expression on CD8 T cells (above the median) with faster progression (HR 1.64 (95% CI 1.04. 2.60); p = 0.034), after adjusting for baseline CD4 T cell count, ART and pVL. The significant Kaplan-Meier analysis for PD-1/Lag-3 co-expression was not supported by Cox models when adjusting for baseline pVL.

### Associations of PD-1, Tim-3 and Lag-3 with T cell memory subsets

**Fig 4:**
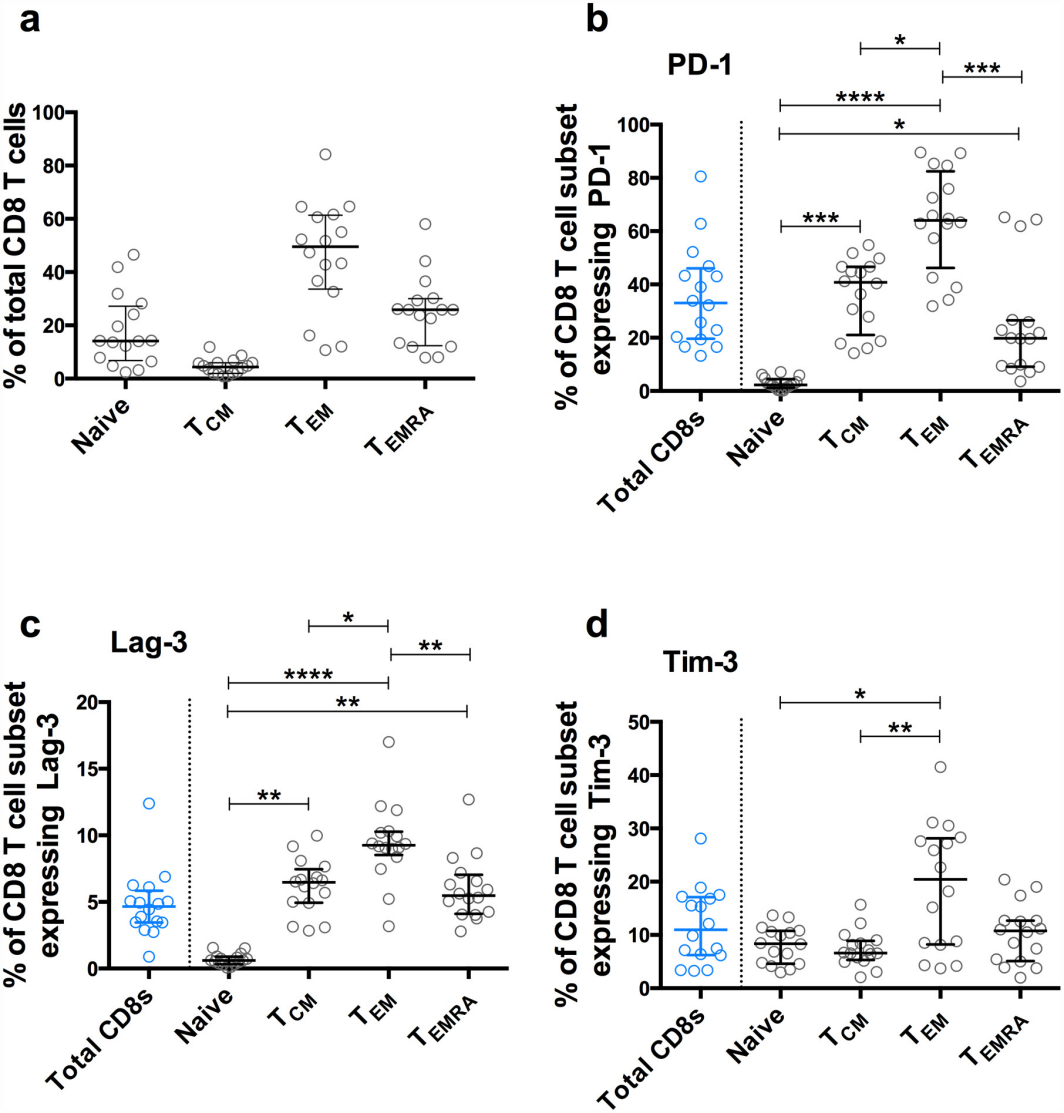
Distribution of PD-1, Lag-3 and Tim-3 expression according to CD8 T cell memory subsets. Overall distribution of naïve, central memory (T_CM_), effector memory (T_EM_) and T_EMRA_ CD8 T cells from pre-therapy baseline samples (a) and percentage expression on each of these subsets at the same time-point of PD-1 (b), Lag-3 (c) and Tim-3 (d). Markers represent medians and interquartile range. *p<0.05, **p<0.01, ***p<0.001, ****p<0.0001. Comparison of exhaustion marker expression across three or more groups was performed using Friedman’s test (non-parametric, paired analysis of variance). Where a difference was found, subsequent pairwise comparisons between groups (Dunn’s test) were performed with adjustment for multiple comparisons targeting on overall significance level of 0.05.

Having determined associations between PD-1, Tim-3 and Lag-3 expression and clinical progression, we next identified the CD8 T cell memory subsets on which these markers were present (Fig E in S1 Text and Fig 4). We analysed samples from 16 participants of a second PHI cohort, HEATHER (‘HIV Reservoir targeting with Early Antiretroviral Therapy’), with similar demographics and inclusion criteria to SPARTAC (Table B in S1 Text). Similar to previous reports in PHI we found that naïve, central memory (T_CM_), effector memory (T_EM_) and T_EMRA_ constituted 14.1, 4.4, 49.6 and 25.9% (median values) of the CD8 T cell population, respectively (Fig 4a). PD-1 and Lag-3 had similar distributions, with significantly higher expression in T_EM_ compared with all other T cell subsets. Although Tim-3 expression was highest amongst T_EM_, there was relatively less expression on T_CM_ compared with PD-1 or Lag-3. Expression of all three markers on naïve cells was very low (especially for PD-1 and Tim-3).

### Functional significance of PD-1, Tim-3 and Lag-3

**Fig 5:**
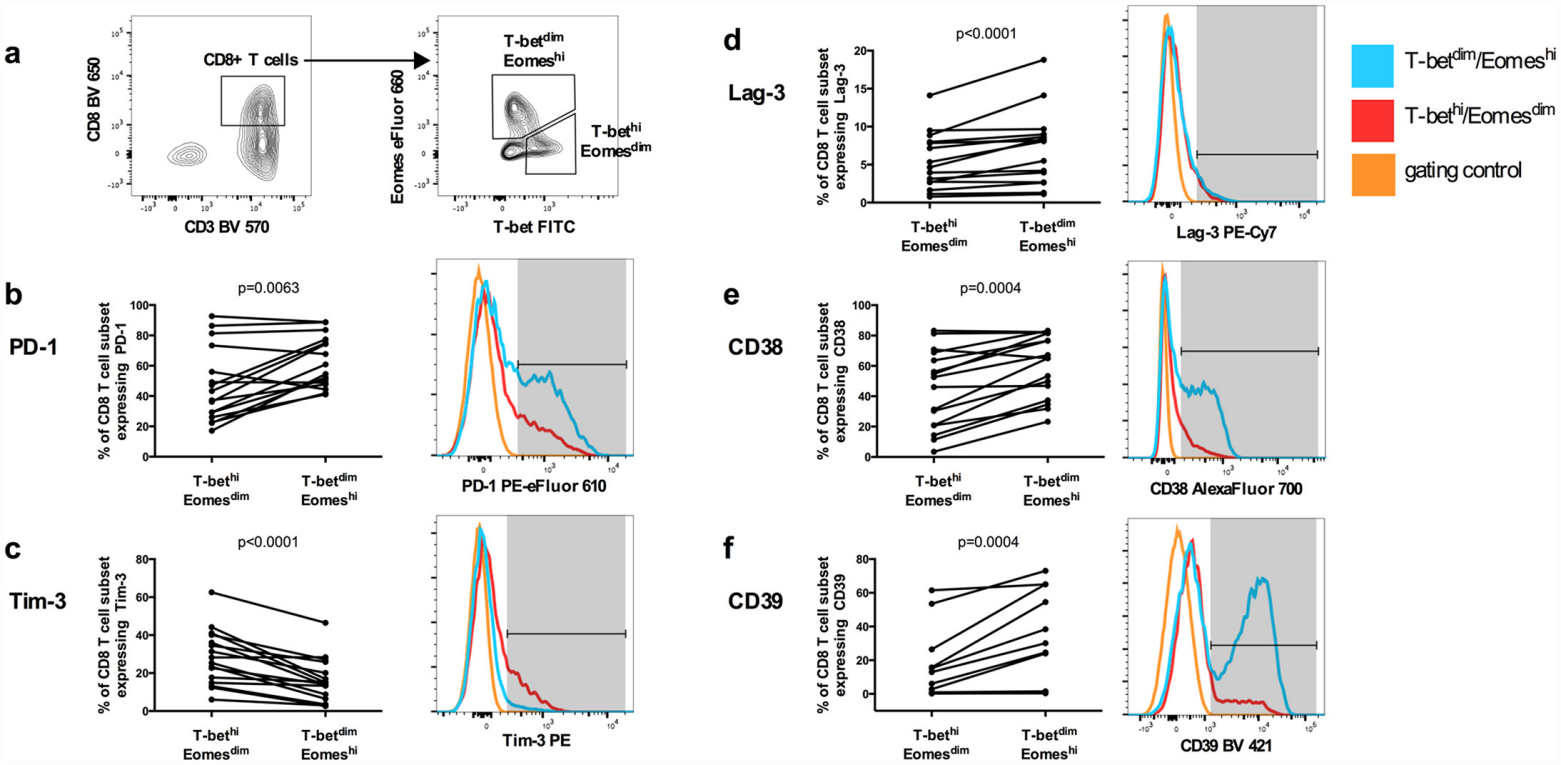
Association between PD-1, Tim-3 or Lag-3 expression on CD8 T cells with T-bet/Eomes and CD39 expression. The strategy for gating CD8 T cells based on T-bet and Eomes expression is shown in (a). Expression of PD-1 (b), Tim-3 (c), Lag-3 (d), CD38 (e) and CD39 (f) was compared between CD8 T cells staining T-bet^hi^/Eomes^dim^ and T-bet^dim^/Eomes^hi^ populations. The panels on the right show the expression of these markers from a single, representative sample with T-bet^hi^/Eomes^dim^ (red line) and T-bet^dim^/Eomes^hi^ (blue line) overlaid with gating control (orange line). The shaded area highlights cells that were considered positive for the marker. Expression of exhaustion markers between two subsets was compared using Wilcoxon matched-pairs signed rank test.

To determine the functional significance of PD-1, Tim-3 and Lag-3 expression in PHI, we compared expression with the T-box transcription factor T-bet, eomesodermin (Eomes) and CD39. In HIV-1 infection, CD8 T cells which are T-bet^dim^/Eomes^hi^ are associated with an exhausted functional phenotype with reduced polyfunctionality[24]. CD39 also identifies exhausted CD8 T cells in HIV-1 infection, and is associated with a transcriptional signature of T cell exhaustion[25]. We, therefore, explored whether these markers were co-expressed with Tim-3, Lag-3 and PD-1 in early HIV-1 infection in the HEATHER cohort. At the nearest available pre-therapy time-point to seroconversion T-bet^dim^/Eomes^hi^ CD8 T cells had significantly higher levels of expression of both PD-1 and Lag-3 (Fig 5). This population also expressed significantly higher levels of CD38 and CD39 but, interestingly, lower levels of Tim-3. We found the expression of CD39 to be bimodal with 50% of participants expressing <1% (Fig F in S1 Text), likely due to polymorphisms in CD39, as recently reported by Roederer et al [26]. There was no evidence for increased co-expression of Tim-3, Lag-3 and PD-1 in the CD39 ‘positive’ (i.e. >1% expression) group compared with those expressing <1%. However, for those individuals with >1% CD39 expression, there was evidence for correlation between levels of CD39 and other exhaustion markers (Fig F in S1 Text).

## Discussion

T cell immune exhaustion has been described as the loss of effector function as a result of repeated antigenic stimulation in persistent infections. Certain cell surface-expressed proteins—also called ‘immune checkpoint receptors’ (ICR)–have been associated with a continuous loss of T cell effector function [17,27], and have attracted much interest as mediators of T cell exhaustion and possible targets for cancer therapies [28–30]. Here, we evaluated the association of ICRs with clinical progression in early HIV-1 infection within the SPARTAC trial. Our results show that ICR expression at the time of enrolment into the study is linked with clinical progression, but that this relationship is highly complex with different ICR markers having a variable impact depending on their co-expression with other exhaustion and activation markers, and as well as the timing of diagnosis of PHI.

Expression of PD-1, Tim-3 and Lag-3 was increased in PHI compared with HIV-uninfected controls and was predominantly found on the CD8 T_EM_ subset. We found strong evidence to support an association between ICR expression and HIV-1 pVL, in line with previous observations [12,20], although not for Tim-3, which contrasts with reports in chronic HIV infection [18]. However, when gated on activated CD38 CD8 T cells, all ICRs correlated strongly with pVL. Expression of CD38 on CD8 T cells is a well-documented marker for HIV-1 disease progression [31] although, surprisingly, we found no evidence supporting this association in our study of primary HIV-1 infection. The increased expression of ICR markers on T_EM_, the association with pVL (and also with CD4 T cell count for PD-1) and the increased strength of the relationship when gated on activated cells are together supportive of a close relationship between exhausted HIV-specific T cell immunity and disease progression. To test this assertion, we turned to primary endpoint data from a randomised controlled trial exploring the impact of short-course ART in PHI on clinical outcomes.

In this sub-analysis of the SPARTAC RCT, there was evidence for PD-1, Lag-3 and Tim-3 predicting disease progression as determined by the time taken to reach the trial primary endpoint (either CD4 T cell count <350 cells/μl or (re)initiation of ART), but the strength of the association varied according to factors such as co-expression with another ICR marker, expression of CD38, and time since seroconversion. Although not evident in the survival analyses, in Cox models only PD-1/Tim-3 co-expression significantly predicted progression when adjusting for CD4 count, ART and pVL.

Despite correlating with pVL, neither Lag-3 nor Tim-3 alone were predictive of clinical progression. Lag-3 is a major histocompatibility complex (MHC) class II ligand that negatively interferes with the positive signal cascade derived from the TCR–MHC interaction. The role of Lag-3 in HIV-1 infection is not yet fully elucidated and data are conflicting[32][33,34]. Although we observed a clear correlation with pVL, sole Lag-3 expression on CD8 and CD38 CD8 T cells did not translate into an association with clinical disease progression.

Tim-3 expression showed distinct associations with clinical disease progression and may even be antagonistic to PD-1 early in HIV-1 infection; Tim-3 was associated with delayed progression in participants identified within 12 weeks of seroconversion, but no effect was seen for Tim-3/PD-1 co-expression. This protective Tim-3 effect was not seen for individuals recruited later than 12 weeks after seroconversion, for whom PD-1/Tim-3 co-expression was disadvantageous. The mechanism of the immuno-regulatory effect of Tim-3 is not clear [35], but this marker has been associated with dysfunction of HIV-1 specific CD8 T cells in chronically infected individuals [18]. Further evidence for the complex role of Tim-3 can be found in the LCMV model where it was only transiently up-regulated during acute infection [36], whereas in chronic infection PD-1/Tim-3 T cells were more exhausted with impaired proliferative capacities than PD-1 single positive T cells [36]. Tim-3 may be crucial in the early phases of HIV-1 infection to ensure the differentiation of antigen specific effector T cells whereas later in infection continuous Tim-3 signalling may lead to T cell exhaustion [35].

Our study has several limitations. First, we evaluated the expression of ICRs on CD8 and CD8 CD38 T cells irrespective of their restriction, although we showed that the majority of ICR expression was on the T_EM_ memory subset. Further in depth longitudinal analyses of these responses in HIV-1 specific T cell populations in conjunction with their activation status will be needed to confirm our findings. Secondly, other investigators have suggested that in acute HIV-1 infection PD-1 expression does not necessarily equate to exhaustion [37]. We could not evaluate the functionality of the immune responses to compare with exhaustion T cell status due to restricted sample availability. However, we demonstrated strong associations of these ICRs during PHI with the T-bet^dim^/Eomes^hi^ CD8 population and CD39 expression. This provides evidence of functional exhaustion of these ICR-expression cells as T-bet^dim^/Eomes^hi^ and CD39 expressing CD8 T cells have been shown to have reduced polyfunctionality, cytokine expression and exhausted transcriptional profiles [24,25,38]. Ideally, one would be able to tease out the overlap between T cell exhaustion and activation—which functionally appear to lie on a similar spectrum. However, the different pathways associated with each ICR marker, as well as their low levels of co-expression, might suggest that the phenotypes are mechanistically separate and that they are independent targets for future therapies and interventions.

The SPARTAC trial consisted of different ART durations and we were not able to show any differences between trial arms, rendering the impact of different ART durations in PHI on the expression of immune exhaustion markers unanswered. Nevertheless, we did correct for the various treatment arms in the multivariable Cox model, and still found significant correlations with exhaustion marker expression and markers of disease progression. Finally, due to the lack of remaining samples from SPARTAC, we turned to a second cohort of individuals sampled during PHI (HEATHER) to explore ICR expression on memory CD8 T cell subsets in conjunction with T-bet, Eomes and CD39. Different antibody clones and fluorochromes were used for these two cohorts and this will have an impact on measured levels of expression, particularly notable for PD-1. However, in none of our analyses do we combine data from the two cohorts and as these two PHI cohorts were very similar in terms of inclusion criteria and demographics, we would expect our findings to be closely related. Of note the characteristics of the HEATHER and SPARTAC participants were comparable, both largely MSM UK infected individuals with B clade virus identified with maximum of 6 months from a previous HIV negative test all starting immediate ART at PHI diagnosis.

In conclusion, our study is the first to show an association between ICR expression and clinical disease progression in a large cohort of 122 HIV-1 seroconverters. However, it is clear that the relationship between T cell immune activation, immune exhaustion, individual ICR markers and clinical outcomes is complex and further studies will be needed to clarify this. Our data suggest that interventions aimed at reversing T cell exhaustion and restoring T cell functionality may be most successful if applied shortly after HIV-1 acquisition, before an exhausted T cell phenotype is established. This may be also be pertinent for enhancing interventions which aim to reactivate the latent HIV reservoir to facilitate immune-directed killing in novel curative HIV strategies.

## Materials and Methods

### Ethics statement

The SPARTAC trial was approved by the following authorities: Medicines and Healthcare products Regulatory Agency (UK), Ministry of Health (Brazil), Irish Medicines Board (Ireland), Medicines Control Council (South Africa), and the Uganda National Council for Science and Technology (Uganda). It was also approved by the following ethics committees in the participating countries: Central London Research Ethics Committee (UK), Hospital Universitário Clementino Fraga Filho Ethics in Research Committee (Brazil), Clinical Research and Ethics Committee of Hospital Clinic in the province of Barcelona (Spain), The Adelaide and Meath Hospital Research Ethics Committee (Ireland), University of Witwatersrand Human Research Ethics Committee, University of Kwazulu-Natal Research Ethics Committee and University of Cape Town Research Ethics Committee (South Africa), Uganda Virus Research Institute Science and ethics committee (Uganda), The Prince Charles Hospital Human Research Ethics Committee and St Vincent's Hospital Human Research Ethics Committee (Australia), and the National Institute for Infectious Diseases Lazzaro Spallanzani, Institute Hospital and the Medical Research Ethics Committee, and the ethical committee Of the Central Foundation of San Raffaele, MonteTabor (Italy). HEATHER (‘HIV Reservoir targeting with Early Antiretroviral Therapy’) was approved by the West Midlands—South Birmingham Research Ethics Committee reference 14/WM/1104. Ethical approvals include use of samples for the studies described. All samples were analysed anonymously.

### Participants and trial design

#### SPARTAC

Blood samples from participants of the SPARTAC trial (EudraCT Number: 2004-000446-20) recruited in the United Kingdom were used in this study. The design of the trial is reported elsewhere [23]. In brief, SPARTAC was an open randomised controlled trial enrolling adults with PHI from 35 sites in Australia, Brazil, Ireland, Italy, South Africa, Spain, Uganda and the UK, from August 2003 to July 2007. Primary HIV-1 infection was defined as: a positive HIV-1 antibody test within 6 months after a negative test (criterion 1), a negative HIV-1 antibody test with a positive RT-PCR assay for HIV-1 RNA (criterion 2), a low level of HIV antibodies (optical density(OD) <0.6) according to a serologic testing algorithm for recent infection (subtype B strain only) (criterion 3), an equivocal HIV-1 antibody test with a repeat test within 2 weeks showing an increase in the level of HIV antibodies (criterion 4), or clinical manifestations of symptomatic HIV-1 seroconversion illness supported by antigen positivity and less than 4 positive bands on Western blot analysis (criterion 5). The time of seroconversion was estimated as the midpoint between the most recent negative or equivocal antibody test and the first positive test for patients who met criterion 1 or 4; as the date of the test for patients who met criterion 2, 3 (if OD ≤0.01) or 5; and as the date of the test −[(OD×150)÷2] days for patients who met criterion 3 (if OD>0.01). Participants were randomised to receive ART for 48 weeks (ART-48), 12 weeks (ART-12) or no therapy (standard of care, SOC). Where ART is included as a co-variate in subsequent analyses it is this duration of randomised therapy rather than the long-term ART commenced as part of the trial primary end-point to which we refer. ‘Baseline’ refers to date of randomisation. The trial primary endpoint was a composite of either a CD4 T cell count <350 cells/μl (>3 months after randomisation and confirmed within 4 weeks) or initiation of long-term ART. We used 12 weeks as the cut-off for the ‘early’ and ‘late’ patients based on the original report which indicated this time-point to be a discriminator of progression rates.[23]

#### HEATHER

Sixteen PHI participants in the HEATHER (‘HIV Reservoir targeting with Early Antiretroviral Therapy’)(West Midlands—South Birmingham Research Ethics Committee reference 14/WM/1104) cohort were selected at random and based on sample availability (Table B in S1 Text). Inclusion criteria for HEATHER were: HIV-1 positive antibody test within 6 months of a HIV-1 negative antibody test, HIV-1 antibody negative with positive PCR (or positive p24 Ag or viral load detectable), RITA (recent incident assay test algorithm) assay result consistent with recent infection, equivocal HIV-1 antibody test supported by a repeat test within 2 weeks showing a rising optical density, having clinical manifestations of symptomatic HIV seroconversion illness supported by antigen positivity. The time of seroconversion was estimated as the midpoint between the most recent negative or equivocal test and the first positive test for those who met relevant criteria and as the date of test for all other participants. For each, the pre-therapy cryopreserved PBMC sample closest to seroconversion was used for analysis of co-expression of PD-1, Tim-3, Lag-3, Eomes, T-bet, CD39 and for CD8 T cell memory subset phenotyping.

### Flow cytometry

Analyses associating markers with disease progression used cryopreserved PBMCs from SPARTAC samples taken at baseline (prior to any ART, if prescribed). Cell surface staining for flow cytometry was performed with: Live/Dead Pacific Blue 2ug/100ul (Invitrogen), CD19 Pacific Blue 0.4 μg/100μl (SJ25-C1), CD3 Pacific Orange 0.4 μg/100μl (UCHT1), CD8 PE-Cy5 PerCP 0.4 μg/100μl (SK1)[BD Bioscience], PD-1 APC 0.5 μg/100μl (MIH4)[eBioscience], Tim-3 PE 0.05 μg/100μl (344823)[R&D], Lag-3 FITC 2 μg/100μl (17B4)[LifeSpan Bioscience] and CD38 PE-Cy7 0.4 μg/100μl (HIT-2)[Biolegend]. The isotype controls for Lag-3 was IgG2a isotype FITC 0.4 μg/100μl (C45)[AdB serotech], for PD-1 was IgG1 isotype APC 0.4 μg/100μl (p3)[eBioscience] and for Tim-3 was IgG2a isotype PE 0.4 μg/100μl (R35-95)[BD Bioscience]. Cell population gating was performed based on mean fluorescence intensity “minus one” (FMO) [39] and unstained controls (Fig A in S1 Text). Samples were acquired on a LSR II (BD) with standard laser configurations and analysed using FlowJo Version 8.7.7.

Analyses examining ICR expression on T cell memory subsets used cryopreserved PBMCs from the HEATHER cohort. Cells were stained in BD Horizon Brilliant Stain Buffer (BD) containing all antibodies and Live/Dead Near IR at 1 in 300 dilution (Life Technologies) in 96 well-V bottom plates at 4°C. PBMCs were stained with the following antibodies: CD3 Brilliant Violet (BV) 570 0.16 μg/100μl (UCHT1), CCR7 Pacific Blue 1.8 μg/100μl (G043H7)[BioLegend], CD4 BV 605 0.05 μg/100μl (RPA-T4), CD8 BV 650 0.012 μg/100μl (RPA-T8)[BD], PD-1 PE eFluor 610 0.2 μg/100μl (eBioJ105), Lag-3 PE-Cy7 0.024 μg/100μl (3D5223H), CD45RA FITC 0.4 μg/100μl (HI100)[eBioscience] and Tim-3 PE (as above).

For characterisation of T-bet/Eomes expression, PBMCs were stained as above in PBS with 5% fetal bovine serum and 1mM EDTA containing Live/Dead Near IR, anti-PD-1, anti-Tim-3, anti-Lag-3, along with antibodies to CD39 BV 421 0.1 μg/100μl (A1) and CD38 AlexaFluor 700 0.1 μg/100μl (HB-7)[BioLegend]. Fixation and permeabilisation were performed with Foxp3 Buffer Set (BD) as per manufacturer’s instructions in reduced volumes to facilitate staining in 96-well plates. Staining of intracellular epitopes was performed at room temperature in PBS containing 0.5% BSA and 0.1% sodium azide with antibodies to CD3, CD4, CD8 (as above), and T-bet FITC 2 μg/100μl (4B10)[BioLegend] and Eomes eFluor660 0.024 μg/100μl (WD1928)[eBioscience]. All samples were acquired on a LSR II (BD). Data were analysed using FlowJo Version 10.8.0r1 (Treestar). Naïve T cells were defined as CD45RA+/CCR7+, T_CM_ as CD45RA-/CCR7+, T_EM_ defined as CD45RA-/CCR7- and T_EMRA_ as CD45RA+/CCR7-. (Fig Ea in S1 Text). CD8 T cells were divided into T-bet^hi^/Eomes^dim^ and T-bet^dim^Eomes^hi^ populations as described by Buggert et al [24](Fig 5a). Gates for exhaustion marker positive populations were set on a partially stained anchor sample without the relevant antibody such that <0.1% of events were positive for the marker (Fig Eb and Ec in S1 Text).

### Statistical methods

Associations between T cell exhaustion markers on CD8 and CD38 CD8 positive cells and pVL or CD4 T cell count were evaluated using Spearman correlations. The fitted line, superimposed in the relevant scatterplots, was estimated through linear regression. When multiple markers were tested for correlations with other variables (e.g. baseline CD4 T cell count or HIV-1 RNA), the adjusted overall critical p-values were also reported. Adjustments were performed using the method of Simes [40] targeting on an overall false discovery rate of ≤0.05. Association of exhaustion markers with clinical progression (time to trial end-point) was assessed by Kaplan-Meier survival curves, log rank tests and Cox proportional hazards models. Covariables considered for all Cox models included ART treatment (as a time updated binary variable), baseline CD4 T cell count and pVL. Effects of the time interval between the estimated date of seroconversion and a baseline exhaustion marker’s measurement (as a binary variable i.e. >12 or ≤12 weeks) and its first order interactions with the aforementioned covariates or the exhaustion markers under investigation were also assessed and, if significant, included in the final multivariable Cox model. Finally, we checked for any effects of the time gap between the last negative and first positive HIV tests used to estimate the seroconversion date. These analyses were performed using Stata 11 (Stata Corp., TX USA). P-values<0.05 were considered as statistically significant.

In the analyses from the HEATHER cohort, comparison of exhaustion marker expression across three or more groups was performed using Friedman’s test (non-parametric, paired analysis of variance). Where a difference was found, subsequent pairwise comparisons between groups (Dunn’s test) were performed with adjustment for multiple comparisons targeting on overall significance level of 0.05. Expression of exhaustion markers between two T cell subsets was compared using Wilcoxon matched-pairs signed rank test. Statistical analyses were performed using GraphPad Prism 6.0f.

## Supporting Information

S1 Text Supporting information. **Table A.** Summary of log rank test results from Fig D in S1 Text. **Table B.** Demographic and clinical characteristics of participants in the HEATHER trial included in the analyses. **Table C.** Correlations of PD-1, Tim-3, Lag-3, PD1/Tim-3, PD1/Lag-3 and Tim-3/Lag-3. **Table D.** Cox Model adjusted for Tim-3, PD-1, Lag-3, baseline CD4 and ART. **Fig A.** Gating strategy: proportion of the total CD8 T cell population that express PD-1, Tim-3, Lag-3 or CD38. **Fig B.** Expression of PD-1, Tim-3 and Lag-3 on CD8 T cells in healthy controls and Primary HIV Infection. **Fig C.** Impact of Tim-3 and Lag-3 expression on CD38 CD8 T cells on clinical outcome. **Fig D.** Impact of co-expression at baseline on CD8 T cells of PD-1, Tim-3 and Lag-3 on clinical outcome. **Fig E.** Gating strategy used for the characterisation of PD-1, Tim-3 and Lag-3 on memory subsets. Fig F. Correlation of CD39 expression with PD-1, Lag-3 and Tim-3.
(DOCX)
