# Supplementary material for: Exhaustion of Activated CD8 T Cells Predicts Disease Progression in Primary HIV-1 Infection
Source: PLoS Pathog. 2016 Jul 14;12(7):e1005661. doi: 10.1371/journal.ppat.1005661 (PMC4945085; doi:10.1371/journal.ppat.1005661)
Supplement: S1 Text — Table A. Summary of log rank test results from Fig D in S1 Text. Table B. Demographic and clinical characteristics of participants in the HEATHER trial included in the analyses. Table C. Correlations of PD-1, Tim-3, Lag-3, PD1/Tim-3, PD1/Lag-3 and Tim-3/Lag-3. Table D. Cox Model adjusted for Tim-3, PD-1, Lag-3, baseline CD4 and ART. Fig A. Gating strategy: proportion of the total CD8 T cell population that express PD-1, Tim-3, Lag-3 or CD38. Fig B. Expression of PD-1, Tim-3 and Lag-3 on CD8 T cells in healthy controls and Primary HIV Infection. Fig C. Impact of Tim-3 and Lag-3 expression on CD38 CD8 T cells on clinical outcome. Fig D. Impact of co-expression at baseline on CD8 T cells of PD-1, Tim-3 and Lag-3 on clinical outcome. Fig E. Gating strategy used for the characterisation of PD-1, Tim-3 and Lag-3 on memory subsets. Fig F. Correlation of CD39 expression with PD-1, Lag-3 and Tim-3. (DOCX) [file ppat.1005661.s001.docx]

**Supplementary Information**

**Table A. Summary of log rank test results from Figure S4**

| Cells studied | ICR Marker | All Participants | Time from seroconversion to baseline ≤ 12 weeks | Time from seroconversion to baseline > 12 weeks | Panel in Supp Fig S4 |
| --- | --- | --- | --- | --- | --- |
| CD8 | PD1/Lag-3 | **0.012** | 0.074 | **0.030** | a-c |
| CD38 CD8 | PD1/Lag-3 | **0.027** | **0.008** | 0.679 | d-f |
|  |  |  |  |  |  |
| CD8 | PD-1/Tim-3 | 0.073* | 0.211 | **0.024** | g-i |
| CD38 CD8 | PD-1/Tim-3 | **0.010** | **0.008** | 0.255 | j-l |
|  |  |  |  |  |  |
| CD8 | Lag-3/Tim-3 | 0.533 | 0.658 | 0.105 | m-o |
| CD38 CD8 | Lag-3/Tim-3 | 0.318 | 0.537 | 0.128 | p-r |

**Table B. Demographic and clinical characteristics of participants in the HEATHER trial included in the analyses**

|  | **N=16** |
| --- | --- |
| **Sex** |  |
| *Male* | 16 (100) |
| *Female* |  |
| **Risk Group** |  |
| *MSM* | 15 (94) |
| *Heterosexual* | - |
| *Unknown* | 1 (6) |
| **Age**  *(years)* | 34 (29, 39) |
| **Baseline CD4 count**  *(cells/μL)* | 553 (408, 669) |
| **Baseline HIV-1 RNA**  *(log_10_ copies/mL)* | 4.9 (4.1, 6.3) |
| **Days from seroconversion to sampling** | 56 (31, 88) |

**Table C. Correlations of PD-1, Tim-3, Lag-3, PD1/Tim-3, PD1/Lag-3 and Tim-3/Lag-3**

|  | **PD-1** | **Tim-3** | **Lag-3** | **PD-1/Tim-3** | **PD-1/Lag-3** |
| --- | --- | --- | --- | --- | --- |
| **Tim-3** | **-0.014** |  |  |  |  |
|  | **0.8781** |  |  |  |  |
|  |  |  |  |  |  |
| **Lag-3** | **0.1955** | **0.0386** |  |  |  |
|  | **0.0309** | **0.6718** |  |  |  |
|  |  |  |  |  |  |
| **PD-1/Tim-3** | **0.4791** | **0.5194** | **0.2503** |  |  |
|  | **<0.001** | **<0.001** | **0.007** |  |  |
|  |  |  |  |  |  |
| **PD-1/Lag-3** | **0.428** | **0.087** | **0.732** | **0.563** |  |
|  | **<0.001** | **0.3554** | **<0.001** | **<0.001** |  |
|  |  |  |  |  |  |
| **Tim-3/Lag-3** | **0.1999** | **0.3432** | **0.7659** | **0.4365** | **0.622** |
|  | **0.0322** | **0.0002** | **<0.001** | **<0.001** | **<0.001** |

**Table D. Cox Model adjusted for Tim-3, PD-1, Lag-3, baseline CD4 and ART**

| Covariate Haz. Ratio Std. Err. P>\|z\| 95% CI |
| --- |
| % CD8+ TIM-3+ Week 0 |
| Below median*                  1 |
| Above median               0.576      0.179     0.075      (0.313 to 1.058) |
| % CD8+ PD-1+  Week 0 |
| Below median*                  1 |
| Above median               1.992      0.586     0.019      (1.120 to 3.545) |
| % CD8+ LAG-3+ Week 0 |
| Below median*                  1 |
| Above median               1.210      0.352     0.512      (0.684 to 2.140) |
| Baseline CD4 count |
| per 100 units              0.752      0.073     0.004      (0.621 to 0.911) |
| Trt |
| 0*                             1 |
| 1                          0.102      0.105     0.027      (0.014 to 0.773) |

**Figure A. Gating strategy: proportion of the total CD8 T cell population that express PD-1, Tim-3, Lag-3 or CD38**


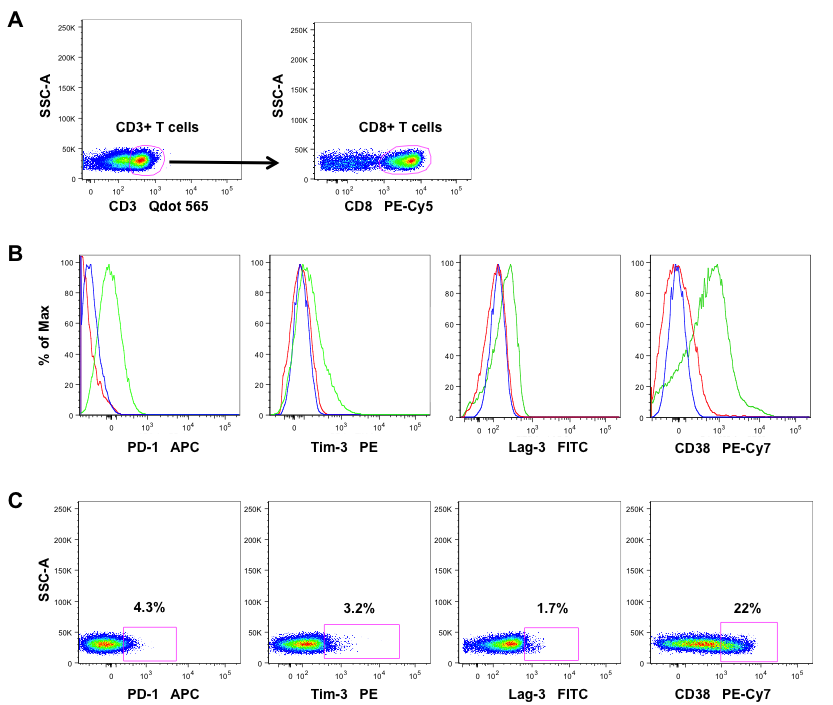


**Figure B. Expression of PD-1, Tim-3 and Lag-3 on CD8 T cells in healthy controls and Primary HIV Infection**

**Figure C.** **Impact of Tim-3 and Lag-3 expression on CD38 CD8 T cells on clinical outcome**

**
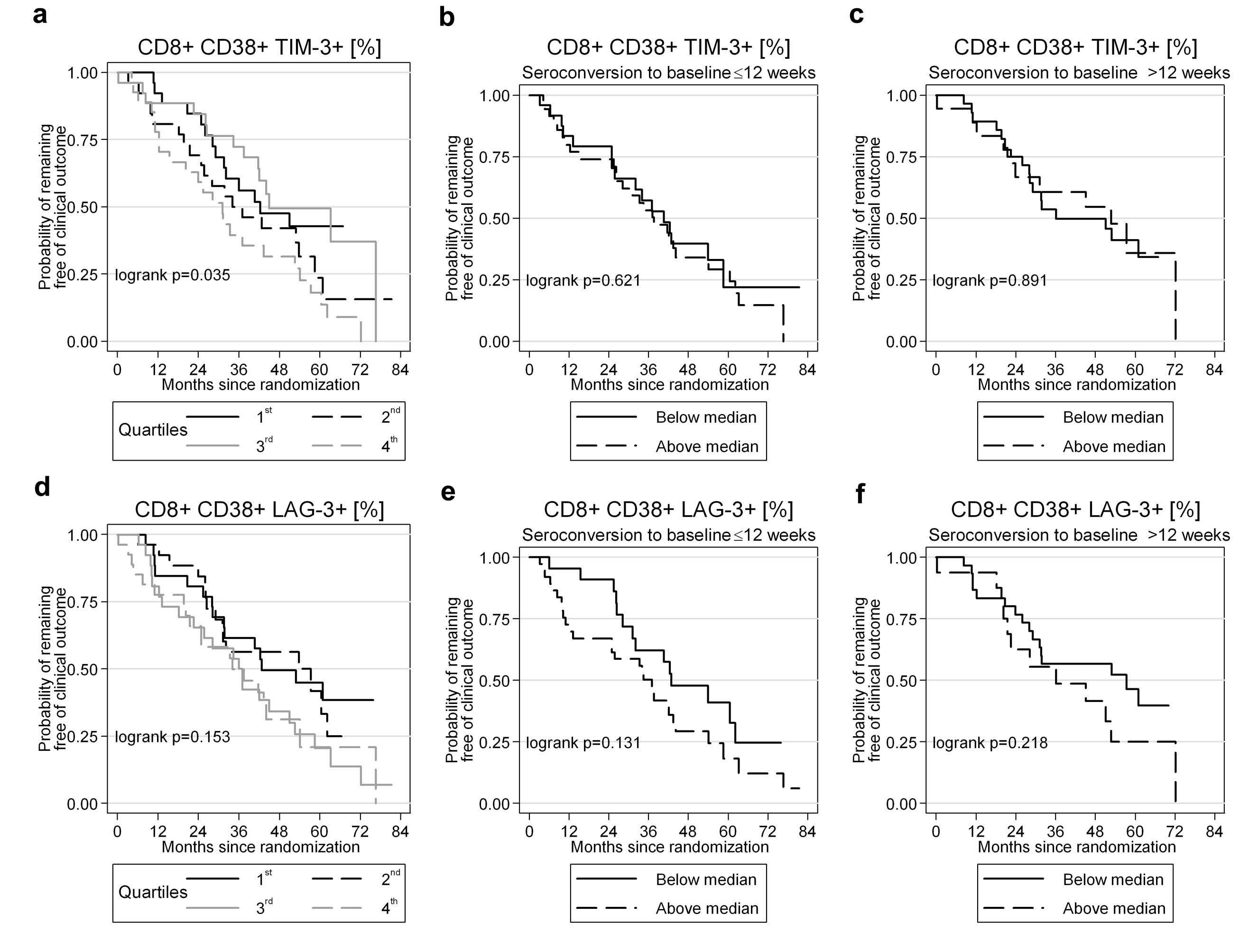
**

**Figure D. Impact of co-expression at baseline on CD8 T cells of PD-1, Tim-3 and Lag-3 on clinical outcome**


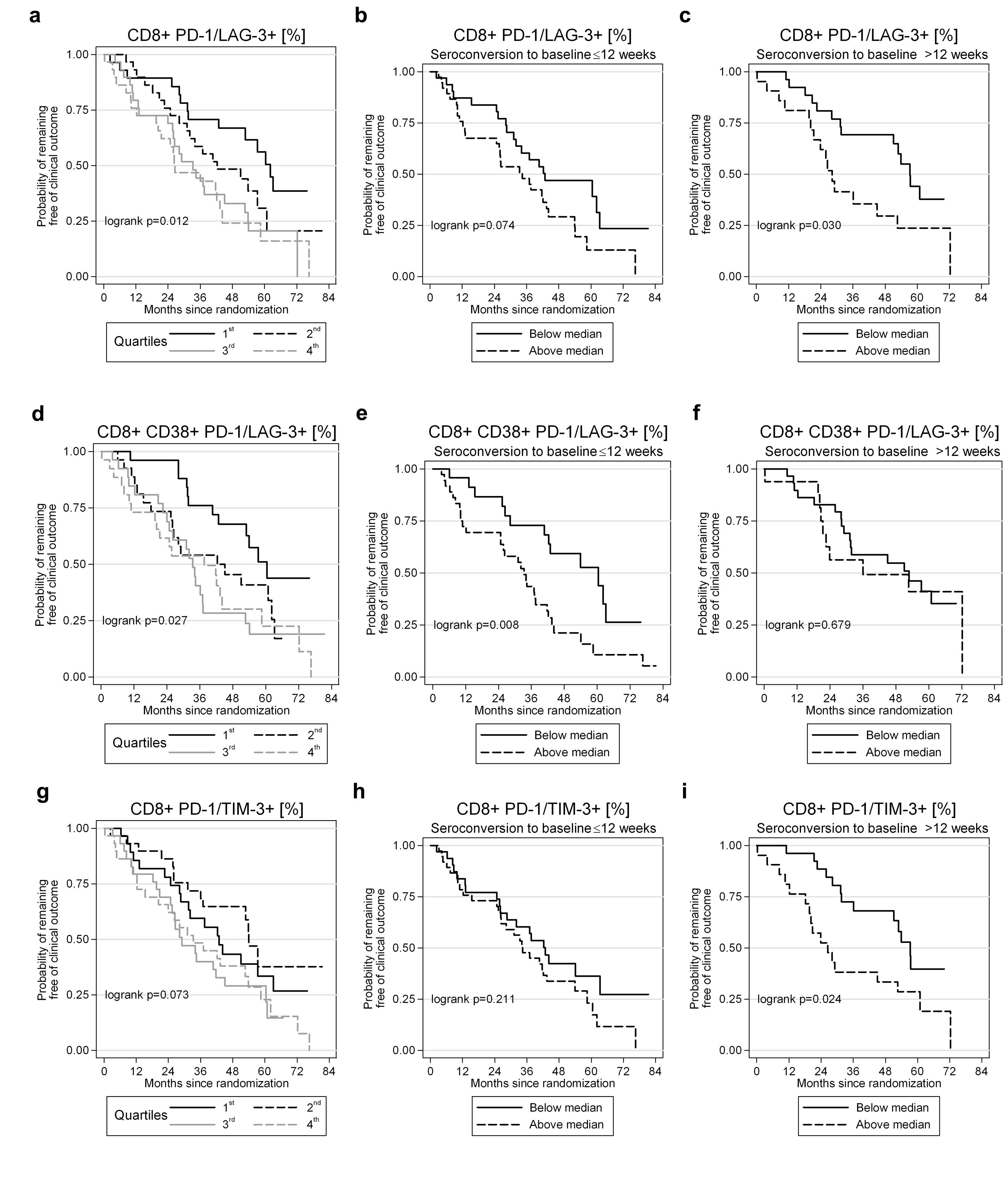


**
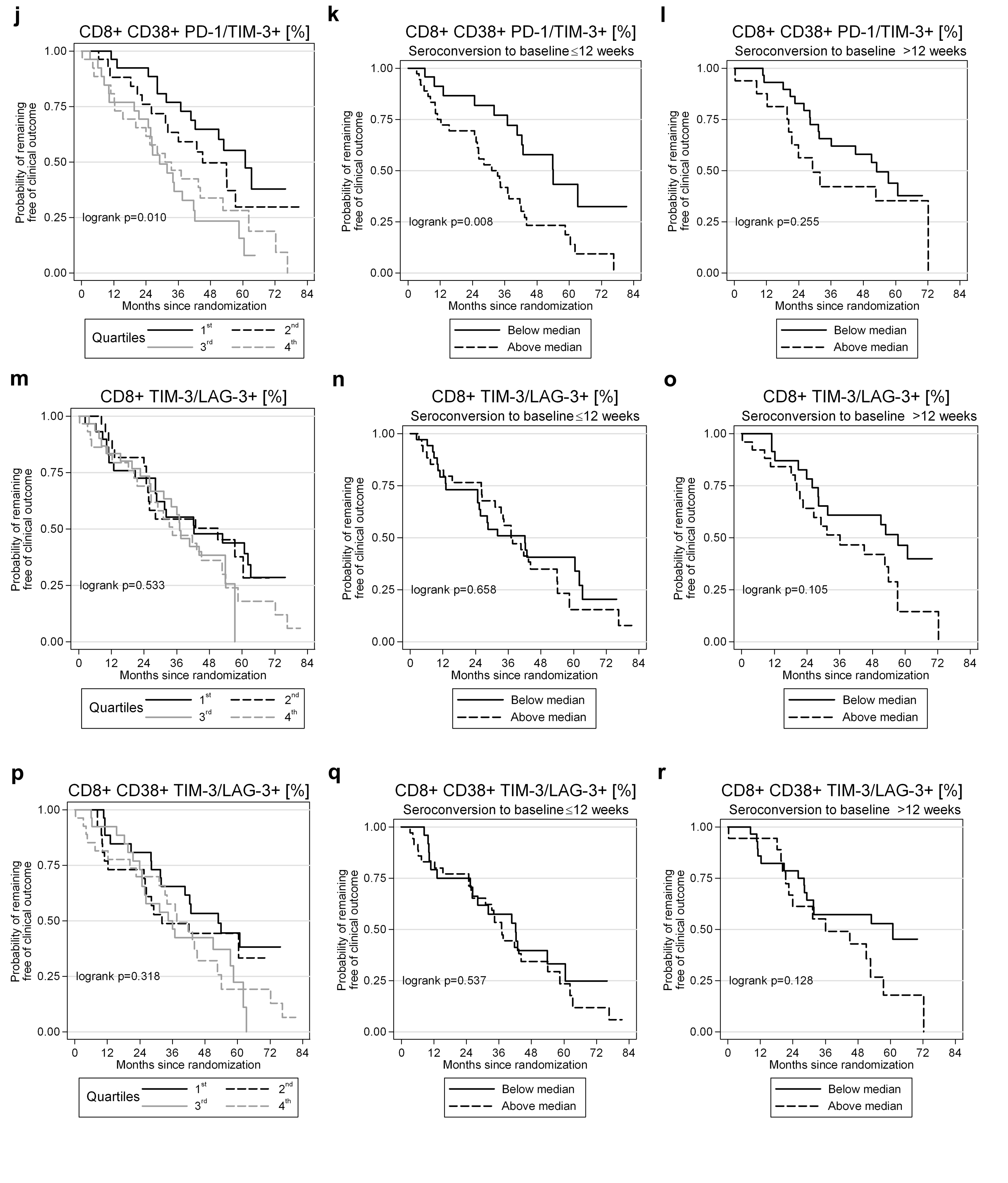
**

**Figure E Gating strategy used for the characterisation of PD-1, Tim-3 and Lag-3 on memory subsets**

**
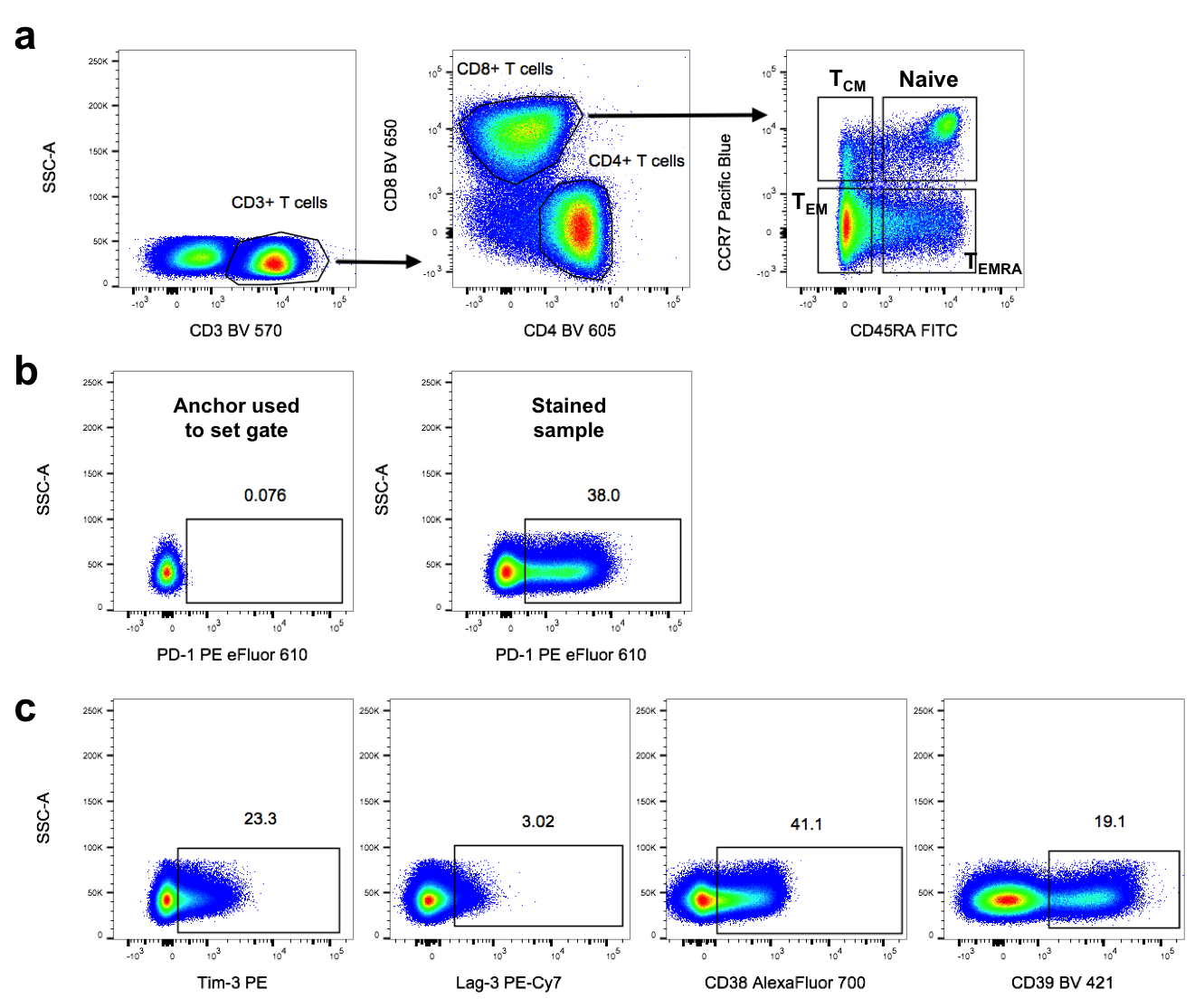
**

**Figure F. Correlation of CD39 expression with PD-1, Lag-3 and Tim-3**


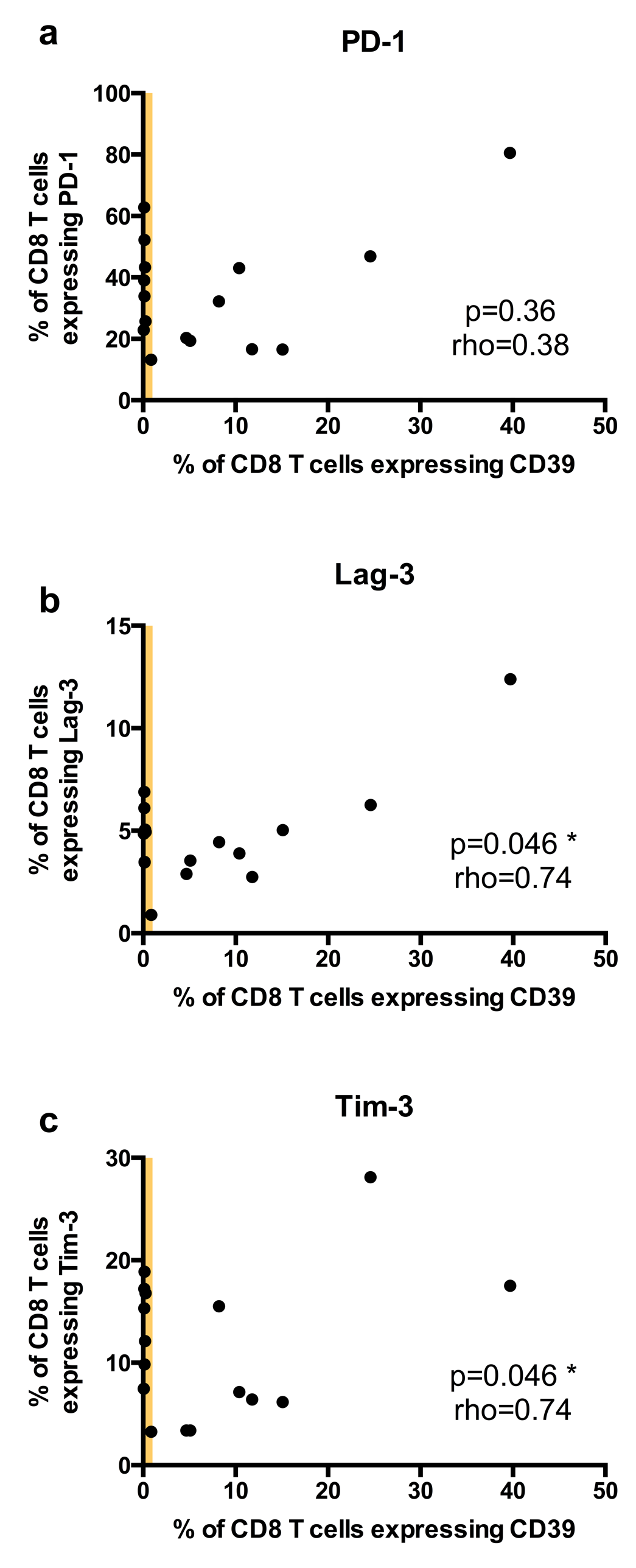


**Legend for Supporting Information**

**Table A. Summary of log rank test results from Figure S4**

Values represent P values derived from the log rank tests, and are not corrected for multiple comparisons. Bold values are those with uncorrected P values <0.05.

* Significant in Cox proportional hazards models when adjusting for baseline CD4 T cell count, ART and pVL.

**Table B. Demographic and clinical characteristics of participants in the HEATHER trial included in the analyses.** Data are presented for the 16 participants from the HEATHER cohort. Values are n(%) for categorical and median (interquartile range) for continuous variables.

**Table C. Correlations of PD-1, Tim-3, Lag-3, PD1/Tim-3, PD1/Lag-3 and Tim-3/Lag-3.** Table to show the correlations between percentage expression of Immune Checkpoint Receptors (for sole and co-expression) on CD8 T cells at the pre-therapy baseline time-point. The first number is Spearman's rho, second is the P value.

**Table D. Cox Model adjusted for Tim-3, PD-1, Lag-3, baseline CD4 and ART.** Cox proportional hazards model adjusted for expression on CD8 T cells of PD-1, Lag-3, Tim-3 as well as baseline CD4 count and ART, to determine associations with the SPARTAC trial primary endpoint.

**Figure A. Gating strategy: proportion of total CD8 T cell population that express PD-1, Tim-3, Lag-3 or CD38.**

Representative gating strategy to identify CD8 T cell populations positive for PD-1, Lag-3, Tim-3 or CD38 expression from the SPARTAC trial. Cryopreserved PBMCs where thawed and subsequently gated on lymphocytes expressing CD3 and CD8 after dead cell and doublet cell exclusion (a). These CD3 and CD8 positive cells were subsequently gated on PD-1, Lag-3, Tim-3 and CD38. Panel (b) shows the identification startegy for these markers: positive CD3 CD8 cells (green) were identified based on an respective isotype control (blue) and ‘fluorescence minus one’ (FMO; red) stains for each suface marker. Panel (c) shows an example of the gating strategy according the positive population identification outlined in panel (b).

**Figure B. Expression of PD-1, Tim-3 and Lag-3 on CD8 T cells in healthy controls and Primary HIV Infection**

Percentage expression on CD8 T cells of PD-1, Lag-3 and Tim-3 is shown for healthy controls and the subjects analysed in this study. P values were calculated using Mann-Whitney tests. The horizontal line within the data represents the median values.

**Figure C.** **Impact of Tim-3 and Lag-3 expression on CD38 CD8 T cells on clinical outcome**

Survival analyses to show impact of co-expression with CD38 for Tim-3 (a-c) or Lag-3 (d-f) on time to the primary end-point in the SPARTAC trial (CD4 T cell count <350 cells/μl or initiation of long-term ART). For Tim-3 data are presented (a) for the entire study group divided into quartiles based on levels of co-expression, (b) for participants recruited within 12 weeks of the estimated date of seroconversion with expression divided at the median and (c) for participants recruited after 12 weeks of the estimated date of seroconversion with expression divided at the median. For Lag-3, data are presented (d) for the entire study group divided into quartiles based on levels of co-expression, (e) for participants recruited within 12 weeks of the estimated date of seroconversion with expression divided at the median and (f) for participants recruited after 12 weeks of the estimated date of seroconversion with expression divided at the median. Significance is tested using a log rank test.

**Figure D. Impact of co-expression at baseline on CD8 T cells of PD-1, Tim-3 and Lag-3 on clinical outcome**

Survival analyses to show impact of co-expression of (a-f) PD-1/Lag-3, (g-l) PD-1/Tim-3, and (m-r) Tim-3/Lag-3 on time to the primary end-point in the SPARTAC trial (CD4 T cell count <350 cells/μl or initiation of long-term ART). For each combination the first three panels (e.g.d-f, j-l, p-r) show additional co-expression with CD38. For all 18 panels, those in the left hand column (a,d,g,j,m,p) show all patients with data presented in quartiles according to expression. The middle panels (b,e,h,k,n,q) and right hand panels (c,f,i,l,o,r) show patients sampled within or after 12 weeks since seroconversion, respectively. Here data are divided at the median expression. Significance is tested using a log rank test.

**Figure E Gating strategy used for the characterisation of PD-1, Tim-3 and Lag-3 on memory subsets**

Representative gating from the analysis performed on samples from the HEATHER cohort. Panel (a) shows the gating strategy used to identify CD8 memory subsets. After dead cell and doublet exclusion, CD3 positive cells were selected and gated based on reciprocal expression on CD4 and CD8. CD8 T cells were then divided into naïve (CD45RA+/CCR7+), Central memory (T_CM_; CD45RA-/CCR7+), effector memory (T_EM_; CD45RA-/CCR7-) and T_EMRA_ (CD45RA+/CCR7-). The approach to setting gates for exhaustion markers is shown in (b). Gates were placed on a partially stained anchor such that <0.1% of cells were positive for marker. An example of this approach is shown for PD-1 on total CD8 T cells. Similar gates for the other markers measured, Tim-3, Lag-3, CD38 and CD39, are shown in panel (c).

**Figure F. Correlation of CD39 expression with PD-1, Lag-3 and Tim-3**

Spearman’s correlations for percentage expression of CD39 on CD8 T cells with (a) PD-1, (b) Lag-3 and (c) Tim-3. The individuals with <1% CD39 expression are shaded in yellow and excluded from the statistical analysis. P-values and Spearman’s rho (2 significant figures) are presented for each including only those participants with >1% CD39 expression.
